# Critical Assessment of Metagenome Interpretation: the second round of challenges

**DOI:** 10.1038/s41592-022-01431-4

**Published:** 2022-04-08

**Authors:** Fernando Meyer, Adrian Fritz, Zhi-Luo Deng, David Koslicki, Till Robin Lesker, Alexey Gurevich, Gary Robertson, Mohammed Alser, Dmitry Antipov, Francesco Beghini, Denis Bertrand, Jaqueline J. Brito, C. Titus Brown, Jan Buchmann, Aydin Buluç, Bo Chen, Rayan Chikhi, Philip T. L. C. Clausen, Alexandru Cristian, Piotr Wojciech Dabrowski, Aaron E. Darling, Rob Egan, Eleazar Eskin, Evangelos Georganas, Eugene Goltsman, Melissa A. Gray, Lars Hestbjerg Hansen, Steven Hofmeyr, Pingqin Huang, Luiz Irber, Huijue Jia, Tue Sparholt Jørgensen, Silas D. Kieser, Terje Klemetsen, Axel Kola, Mikhail Kolmogorov, Anton Korobeynikov, Jason Kwan, Nathan LaPierre, Claire Lemaitre, Chenhao Li, Antoine Limasset, Fabio Malcher-Miranda, Serghei Mangul, Vanessa R. Marcelino, Camille Marchet, Pierre Marijon, Dmitry Meleshko, Daniel R. Mende, Alessio Milanese, Niranjan Nagarajan, Jakob Nissen, Sergey Nurk, Leonid Oliker, Lucas Paoli, Pierre Peterlongo, Vitor C. Piro, Jacob S. Porter, Simon Rasmussen, Evan R. Rees, Knut Reinert, Bernhard Renard, Espen Mikal Robertsen, Gail L. Rosen, Hans-Joachim Ruscheweyh, Varuni Sarwal, Nicola Segata, Enrico Seiler, Lizhen Shi, Fengzhu Sun, Shinichi Sunagawa, Søren Johannes Sørensen, Ashleigh Thomas, Chengxuan Tong, Mirko Trajkovski, Julien Tremblay, Gherman Uritskiy, Riccardo Vicedomini, Zhengyang Wang, Ziye Wang, Zhong Wang, Andrew Warren, Nils Peder Willassen, Katherine Yelick, Ronghui You, Georg Zeller, Zhengqiao Zhao, Shanfeng Zhu, Jie Zhu, Ruben Garrido-Oter, Petra Gastmeier, Stephane Hacquard, Susanne Häußler, Ariane Khaledi, Friederike Maechler, Fantin Mesny, Simona Radutoiu, Paul Schulze-Lefert, Nathiana Smit, Till Strowig, Andreas Bremges, Alexander Sczyrba, Alice Carolyn McHardy

**Affiliations:** 1 Computational Biology of Infection Research, Helmholtz Centre for Infection Research, Braunschweig, Germany; 2 Braunschweig Integrated Centre of Systems Biology (BRICS), Technische Universität Braunschweig, Braunschweig, Germany; 3 German Center for Infection Research (DZIF), Hannover-Braunschweig Site, Braunschweig, Germany; 4 Cluster of Excellence RESIST (EXC 2155), Hannover Medical School, Hannover, Germany; 5 Pennsylvania State University, State College, PA USA; 6 Helmholtz Centre for Infection Research, Braunschweig, Germany; 7 Saint Petersburg State University, Saint Petersburg, Russia; 8 Department of Information Technology and Electrical Engineering, ETH Zürich, Zurich, Switzerland; 9 Center for Algorithmic Biotechnology, Saint Petersburg State University, Saint Petersburg, Russia; 10 Department CIBIO, University of Trento, Trento, Italy; 11 Genome Institute of Singapore, Singapore, Singapore; 12 University of Southern California, Los Angeles, CA USA; 13 University of California, Davis, Davis, CA USA; 14 Institute for Biological Data Science, Heinrich-Heine-University, Düsseldorf, Germany; 15 Lawrence Berkeley National Laboratory, Berkeley, CA USA; 16 University of California, Berkeley, Berkeley, CA USA; 17 Institut Pasteur, Paris, France; 18 National Food Institute, Division of Global Surveillance, Technical University of Denmark, Lyngby, Denmark; 19 Drexel University, Philadelphia, PA USA; 20 Google Inc., Philadelphia, PA USA; 21 Robert Koch-Institut, Berlin, Germany; 22 Hochschule für Technik und Wirtschaft Berlin, Berlin, Germany; 23 University of Technology Sydney, Sydney, Australia; 24 DOE Joint Genome Institute, Berkeley, CA USA; 25 Lawrence Berkeley National Laboratories, Berkeley, CA USA; 26 University of California, Los Angeles, Los Angeles, CA USA; 27 Intel Corporation, Santa Clara, CA USA; 28 Ecological and Evolutionary Signal-Processing and Informatics Laboratory, Philadelphia, PA USA; 29 University of Copenhagen, Department of Plant and Environmental Science, Frederiksberg, Denmark; 30 School of Computer Science, Fudan University, Shanghai, China; 31 BGI-Shenzhen, Shenzhen, China; 32 Shenzhen Key Laboratory of Human Commensal Microorganisms and Health Research, BGI-Shenzhen, Shenzhen, China; 33 Technical University of Denmark, Novo Nordisk Foundation Center for Biosustainability, Lyngby, Denmark; 34 Aarhus University, Department of Environmental Science, Roskilde, Denmark; 35 Department of Cell Physiology and Metabolism, Faculty of Medicine, University of Geneva, Geneva, Switzerland; 36 Swiss Institute of Bioinformatics, Geneva, Switzerland; 37 The Arctic University of Norway, Tromsø, Norway; 38 Charité—Universitätsmedizin Berlin, Berlin, Germany; 39 Department of Computer Science and Engineering, University of California San Diego, San Diego, CA USA; 40 Department of Statistical Modelling, Saint Petersburg State University, Saint Petersburg, Russia; 41 University of Wisconsin—Madison, Madison, WI USA; 42 Univ. Rennes, Inria, CNRS, IRISA, Rennes, France; 43 Université Lille, CNRS, CRIStAL, Lille, France; 44 Hasso Plattner Institute, Digital Engineering Faculty, University of Potsdam, Potsdam, Germany; 45 Sydney Medical School, The University of Sydney, Sydney, Australia; 46 Centre for Innate Immunity and Infectious Diseases, Hudson Institute of Medical Research, Clayton, Australia; 47 Department of Computer Science, Inria, University of Lille, CNRS, Lille, France; 48 Amsterdam University Medical Center, Amsterdam, the Netherlands; 49 Department of Biology, Institute of Microbiology and Swiss Institute of Bioinformatics, ETH Zürich, Zürich, Switzerland; 50 Structural and Computational Biology Unit, EMBL, Heidelberg, Germany; 51 Genome Institute of Singapore, A*STAR, Singapore, Singapore; 52 National University of Singapore, Singapore, Singapore; 53 DTU Health Tech, Kongens, Lyngby, Denmark; 54 Genome Informatics Section, Computational and Statistical Genomics Branch, National Human Genome Research Institute, National Institutes of Health, Bethesda, MD USA; 55 University of Virginia, Charlottesville, VA USA; 56 Novo Nordisk Foundation Center for Protein Research, Faculty of Health and Medical Sciences, University of Copenhagen, Copenhagen, Denmark; 57 Institute for Bioinformatics, FU Berlin, Berlin, Germany; 58 Bioinformatics Unit (MF1), Robert Koch Institute, Berlin, Germany; 59 Center for Biological Discovery from Big Data, Philadelphia, PA USA; 60 Florida Polytechnic University, Lakeland, FL USA; 61 Quantitative and Computational Biology Department, University of Southern California, Los Angeles, CA USA; 62 University of Copenhagen, Copenhagen, Denmark; 63 University of British Columbia, Vancouver, British Columbia Canada; 64 Diabetes Center, Faculty of Medicine, University of Geneva, Geneva, Switzerland; 65 Energy, Mining and Environment, National Research Council Canada, Montreal, Quebec Canada; 66 Phase Genomics, Seattle, WA USA; 67 School of Mathematical Sciences, Fudan University, Shanghai, China; 68 Department of Energy Joint Genome Institute, Berkeley, CA USA; 69 Environmental Genomics and Systems Biology Division, Lawrence Berkeley National Laboratory, Berkeley, CA USA; 70 School of Natural Sciences, University of California at Merced, Merced, CA USA; 71 Institute of Science and Technology for Brain-Inspired Intelligence, Fudan University, Shanghai, China; 72 Key Laboratory of Computational Neuroscience and Brain-Inspired Intelligence (Fudan University), Ministry of Education, Shanghai, China; 73 Max Planck Institute for Plant Breeding Research, Köln, Germany; 74 Aarhus University, Aarhus, Denmark; 75 Center for Biotechnology (CeBiTec), Bielefeld University, Bielefeld, Germany

**Keywords:** Classification and taxonomy, Metagenomics, Software, Metagenomics, Metagenomics

## Abstract

Evaluating metagenomic software is key for optimizing metagenome interpretation and focus of the Initiative for the Critical Assessment of Metagenome Interpretation (CAMI). The CAMI II challenge engaged the community to assess methods on realistic and complex datasets with long- and short-read sequences, created computationally from around 1,700 new and known genomes, as well as 600 new plasmids and viruses. Here we analyze 5,002 results by 76 program versions. Substantial improvements were seen in assembly, some due to long-read data. Related strains still were challenging for assembly and genome recovery through binning, as was assembly quality for the latter. Profilers markedly matured, with taxon profilers and binners excelling at higher bacterial ranks, but underperforming for viruses and Archaea. Clinical pathogen detection results revealed a need to improve reproducibility. Runtime and memory usage analyses identified efficient programs, including top performers with other metrics. The results identify challenges and guide researchers in selecting methods for analyses.

## Abstract (web summary)

This study presents the results of the second round of the Critical Assessment of Metagenome Interpretation challenges (CAMI II), which is a community-driven effort for comprehensively benchmarking tools for metagenomics data analysis.

## Main

Over the last two decades, advances in metagenomics have vastly increased our knowledge of the microbial world and intensified development of data analysis techniques^1–3^. This created a need for unbiased and comprehensive assessment of these methods, to identify best practices and open challenges in the field^4–7^. CAMI, the Initiative for the Critical Assessment of Metagenome Interpretation, is a community-driven effort addressing this need, by offering comprehensive benchmarking challenges on datasets representing common experimental settings, data generation techniques and environments in microbiome research. In addition to its open and collaborative nature, data FAIRness and reproducibility are defining principles^8^.

The first CAMI challenge^4^ delivered insights into the performances of metagenome assembly, genome and taxonomic binning and profiling programs across multiple complex benchmark datasets, including unpublished genomes with different evolutionary divergences and poorly categorized taxonomic groups, such as viruses. The robustness and high accuracy observed for binning programs in the absence of strain diversity supported their application to large-scale data from various environments, recovering thousands of metagenome-assembled genomes^9,10^ and intensified efforts in advancing strain-resolved assembly and binning. We here describe the results of the second round of CAMI challenges^11^, in which we assessed program performances and progress on even larger and more complex datasets, including long-read data and further performance metrics such as runtime and memory use.

## Results

We created metagenome benchmark datasets representing a marine, a high strain diversity environment (‘strain-madness’) and a plant-associated environment including fungal genomes and host plant material. Datasets included long and short reads sampled from 1,680 microbial genomes and 599 circular elements (Methods and Supplementary Table 1). Of these, 772 genomes and all circular elements were newly sequenced and distinct from public genome sequence collections (new genomes), and the remainder were high-quality public genomes. Genomes with an average nucleotide identity (ANI) of less than 95% to any other genome were classified as ‘unique’, and as ‘common’ otherwise, as in the first challenge^4^. Overall, 901 genomes were unique (474 marine, 414 plant-associated, 13 strain-madness), and 779 were common (303 marine, 81 plant-associated, 395 strain-madness). On these data, challenges were offered for assembly, genome binning, taxonomic binning and profiling methods, which opened in 2019 and 2020 and allowed submissions for several months (Methods). In addition, a pathogen detection challenge was offered, on a clinical metagenome sample from a critically ill patient with an unknown infection. Challenge participants were encouraged to submit reproducible results by providing executable software with parameter settings and reference databases used. Overall, 5,002 results for 76 programs were received from 30 teams (Supplementary Table 2).

### Assembly challenge

Sequence assemblies are key for metagenome analysis and used to recover genome and taxon bins. Assembly quality degrades for genomes with low evolutionary divergences, resulting in consensus or fragmented assemblies^12,13^. Due to their relevance for understanding microbial communities^14,15^, we assessed methods’ abilities to assemble strain-resolved genomes, using long- and short-read data (Methods).

### Overall trends

We evaluated 155 submissions for 20 assembler versions, including some with multiple settings and data preprocessing options (Supplementary Table 2). In addition, we created gold standard co- and single-sample assemblies as in refs. ^4,16^. The gold standards of short, long and hybrid marine data comprise 2.59, 2.60 and 2.79 gigabases (Gb) of assembled sequences, respectively, while the strain-madness gold standards consist of 1.45 Gb each.

**Fig. 1:**
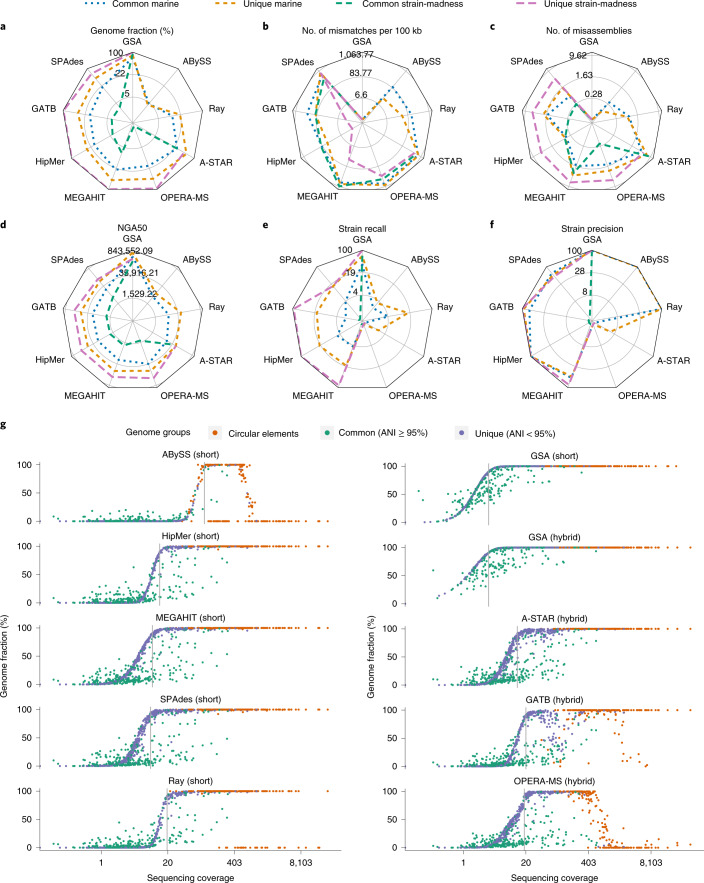
Metagenome assembler performances on the marine and strain-madness datasets. **a**, Radar plots of genome fraction. **b**, Mismatches per 100 kilobases (kb). **c**, Misassemblies. **d**, NGA50. **e**, Strain recall. **f**, Strain precision. For methods with multiple evaluated versions, the best ranked version on the marine data is shown (Supplementary Fig. 1 and Supplementary Table 3). Absolute values for metrics are log scaled. Lines indicate different subsets of genomes analyzed, and the value of the GSAs indicates the upper bound for a metric. The metrics are shown for both unique and common strain genomes. **g**, Genome recovery fraction versus genome sequencing depth (coverage) on the marine dataset. Blue indicates unique genomes (<95% ANI), green common genomes (ANI ≥ 95%) and orange high-copy circular elements. Gray lines indicate the coverage at which the first genome is recovered with ≥90% genome fraction. Source data

Assemblies were evaluated with MetaQUAST v.5.1.0rc (ref. ^17^), adapted for assessing strain-resolved assembly (Supplementary Text). We determined strain recall and precision, similar to ref. ^18^ (Methods and Supplementary Table 3). To facilitate comparisons, we ranked assemblies produced with different versions and parameter settings for a method based on key metrics (Methods) and chose the highest-ranking as the representative (Fig. 1, Supplementary Fig. 1 and Supplementary Tables 3–7).

Short-read assemblers achieved genome fractions of up to 10.4% on strain-madness and 41.1% on marine data, both by MEGAHIT^19^. The gold standard reported 90.8 and 76.9%, respectively (Fig. 1a and Supplementary Table 3). HipMer^20^ ranked best across metrics and datasets, and on marine data, as it produced few mismatches with a comparably high genome fraction and NGA50 (Table 1). On strain-madness data, GATB^21,22^ ranked best, with HipMer in second place. On the plant-associated dataset, HipMer again ranked best, followed by Flye v.2.8 (ref. ^23^), which outperformed other short-read assemblers in most metrics (Supplementary Fig. 2).

**Table 1:** Best ranked software for four categories across datasets, in presence or absence of strain diversity and by computational requirements

	Assembly	Genome binning	Taxon binning	Taxon profiling
Metrics	Strain recall and precision, mismatches per 100 kb, duplication ratio, misassemblies, genome fraction, NGA50	Average completeness and purity, ARI, percentage of binned base pairs	Average completeness and purity, F1-score, accuracy	Completeness, purity, F1-score, L1- norm error, Bray–Curtis, Shannon equitability, weighted UniFrac error
**Best methods across metrics**				
Marine	HipMer 1.0, metaSPAdes 3.13.1, ABySS 2.1.5 (all on SR)	MetaBinner 1.0, UltraBinner 1.0, MetaWRAP 1.2.3	Kraken 2.0.8 beta (GSA), Ganon 0.1.4 (SR), MEGAN 6.15.2 (GSA)	mOTUs 2.5.1, MetaPhlAn 2.9.22 and v.cami1
Strain-madness	GATB 1.0 (hybrid), HipMer 1.0 (SR), OPERA-MS 0.8.3 (hybrid)	CONCOCT 0.4.1, MetaBinner 1.0, UltraBinner 1.0	PhyloPythiaS+ 1.4 (GSA), Kraken 2.0.8 beta (GSA), LSHVec (GSA)	mOTUs v.cami1, MetaPhlAn 2.9.22, DUDes v.cami1
Plant-associated	MetaHipMer 2.0.1.2 (SR), metaFlye 2.8.1 (hybrid), metaSPAdes 3.13.1 (SR)	CONCOCT 0.4.1 and 1.1.0, MaxBin 2.2.7	MEGAN 6.15.2 (GSA), Ganon 0.3.1 (SR), DIAMOND 0.9.28 (GSA)	mOTUs 2.5.1, MetaPhlAn 2.9.21, Bracken 2.6
Strain diversity	GATB 1.0 (hybrid), HipMer 1.0	CONCOCT 0.4.1	PhyloPythiaS+ 1.4 (on strain-madness data)	NA
No strain diversity	HipMer 1.0	UltraBinner 1.0	NA	NA
Fastest	MetaHipMer 2.0.1.2, MEGAHIT 1.2.7	MetaBAT 2.13, Vamb fa045c0	Kraken 2.0.8 beta (GSA, SR), DIAMOND (GSA)	FOCUS 1.5, Bracken 2.2
Most memory efficient	MEGAHIT 1.2.7, GATB 1.0	MetaBAT 2.13, MaxBin 2.0.2	Kraken 2.0.8 beta (GSA, SR), DIAMOND (GSA)	FOCUS 1.5, mOTUs 1.1.1

GSA denotes run on contigs of the GSAs and SR run on short reads. Submission deadlines for the different method categories and datasets are provided in the Methods. The numbers given are the software version numbers.

The best hybrid assembler, A-STAR, excelled in genome fraction (44.1% on marine, 30.9% on strain-madness), but created more misassemblies and mismatches (773 mismatches per 100 kb on marine) than others. HipMer had the fewest mismatches (67) per 100 kb on the marine and GATB on the strain-madness data (98, Fig. 1b). GATB introduced the fewest mismatches (173) among hybrid assemblers on the marine dataset. ABySS^24^ created the fewest misassemblies for the marine and GATB for the strain-madness data (Fig. 1c). The hybrid assembler OPERA-MS^25^ created the most contiguous assemblies for the marine data (Fig. 1d), with an average NGA50 of 28,244 across genomes, compared to 682,777 for the gold standard. The SPAdes^26^ hybrid submission had a higher NGA50 of 43,014, but was not the best ranking SPAdes submission. A-STAR had the highest contiguity for the strain-madness data (13,008 versus 155,979 for gold standard). For short-read assembly, MEGAHIT had the highest contiguity on the marine (NGA50 26,599) and strain-madness data (NGA50 4,793). Notably, Flye performed well on plant-associated long-read data but worse than others across most metrics on the marine data (Supplementary Fig. 2), likely due to different versions or parameter settings (Supplementary Table 2).

For several assemblers, preprocessing using read quality trimming or error correction software, such as trimmomatic^27^ or DUK^28^, improved assembly quality (Supplementary Tables 2 and 3). Genome coverage was also a key factor (Fig. 1g). While gold standards for short and hybrid assemblies included genome assemblies with more than 90% genome fraction and 3.3× coverage, SPAdes best assembled low coverage marine genomes, starting at 9.2×. MEGAHIT, A-STAR, HipMer and Ray Meta^29^ required 10×, 13.2×, 13.9× and 19.5× coverage, respectively. Several assemblers reconstructed high-copy circular elements well, with HipMer, MEGAHIT, SPAdes and A-STAR reconstructing all (Fig. 1g). Compared to software assessed in the first CAMI challenge, A-STAR had a 20% higher genome fraction on strain-madness data, almost threefold that of MEGAHIT. HipMer introduced the fewest mismatches (67 mismatches per 100 kb) on the marine data. This was 30% less than Ray Meta, the best performing method also participating in CAMI 1. OPERA-MS improved on MEGAHIT in NGA50 by 1,645 (6%), although using twice as much (long- and short-read) data. SPAdes, which was not assessed in the first challenge, was among the top submissions for most metrics.

### Closely related genomes

The first CAMI challenge revealed substantial differences in assembly quality between unique and common strain genomes^4^. Across metrics, datasets and software results, unique genome assemblies again were superior, for marine genomes by 9.7% in strain recall, 19.3% genome fraction, sevenfold NGA50 and 6.5% strain precision, resulting in more complete and less fragmented assemblies (Fig. 1 and Supplementary Tables 4–7). This was even more pronounced on the strain-madness dataset, with a 79.1% difference in strain recall, 75.9% genome fraction, 20.6% strain precision and 50-fold NGA50. Although there were more misassemblies for unique than for common genomes (+1.5 in marine, +5.4 in strain-madness), this was due to the larger assembly size of the former, evident by a similar fraction of misassembled contigs (2.6% for unique genomes, 3.1% for common). While the duplication ratio was similar for unique and common genomes (+0.01 marine, −0.08 strain-madness), unique marine genome assemblies had 12% more mismatches than common ones (548 versus 486 mismatches per 100 kb). In contrast, there were 62% fewer mismatches for unique than common strain-madness genome assemblies (199 mismatches per 100 kb versus 511 mismatches per 100 kb), likely due to the elevated strain diversity.

For common marine genomes, HipMer ranked best across metrics and GATB for common strain-madness genomes. On unique genomes, HipMer ranked first for the marine and strain-madness datasets. HipMer had the highest strain recall and precision for common and unique marine genomes (4.5 and 20.4% recall, 100% precision each). For the strain-madness dataset, A-STAR had the highest strain recall (1.5%) on common strain-madness genomes, but lower precision (23.1%). GATB, HipMer, MEGAHIT and OPERA-MS assembled unique genomes with 100% recall and precision. A-STAR excelled in genome fraction, ranking first across all four data partitions and HipMer had the fewest mismatches. HipMer also had the fewest misassemblies on the common and unique marine genomes, while GATB had the fewest misassemblies on common strain-madness genomes and SPAdes on unique ones. The highest NGA50 on common marine genomes was achieved by OPERA-MS, on common strain-madness genomes by A-STAR and on unique genomes in both datasets by SPAdes.

### Difficult to assemble regions

We assessed assembly performances for difficult to assemble regions, such as repeats or conserved elements (for example, 16S ribosomal RNA genes) on high-quality public genomes included in the marine data. These regions are important for genome recovery, but often missed^30^. We selected 50 unique, public genomes with annotated 16S sequences and present as a single contig in the gold standard assembly (GSA). We mapped assembly submissions to these 16S sequences using Minimap2 (ref. ^31^) and measured their completeness (% genome fraction) and divergence^31^ (Supplementary Fig. 3a,b,e). A-STAR partially recovered 102 (78%) of 131 16S sequences. The hybrid assemblers GATB (mean completeness 60.1%) and OPERA-MS (mean 47.1%) recovered the most complete 16S sequences. Mean completeness for short-read assemblies ranged from 29.6% (HipMer) to 36.9% (MEGAHIT). Assemblies were very accurate for ABySS and HipMer (<1% divergence). The hybrid assemblers GATB and OPERA-MS produced the longest contigs aligning to 16S rRNA genes, with a median length of 8,513 and 4,430 base pairs (bp), respectively, while for other assemblers median contig length was less than the average 16S rRNA gene length (1,503 bp). For all assemblers and 16S sequences, there were 17 cross-genome chimeras, reported by MetaQUAST as interspecies translocations: ten for MEGAHIT, five for A-STAR and one each for HipMer and SPAdes, while GATB, ABySS and OPERA-MS did not produce chimeric sequences. We performed the same evaluation for CRISPR cassettes found in 30 of the 50 genomes using different methods^32–34^. CRISPR cassette regions were easier to assemble, as evident by a higher (5–50%) completeness and longer assembled CRISPR-carrying contigs (up to 22× median length) than for 16S rRNA genes (Supplementary Fig. 3c,d,f). Across assemblies and methods, average assembly quality was better for public than for new genomes in key metrics, such as genome fraction and NGA50 (Supplementary Fig. 4).

### Single versus coassembly

For multi-sample metagenome datasets, common assembly strategies are pooling samples (coassembly) and single-sample assembly^10,20,35^. We evaluated the assembly quality for both strategies using genomes spiked into the plant-associated data with specific coverages (Supplementary Table 8) across results for five assemblers (Supplementary Fig. 5). Only HipMer recovered a unique genome split across 16 samples from pooled samples, while a unique, single-sample genome was reconstructed well by all assemblers with both strategies. For genomes unique to a single sample, but common in pooled samples (LjRoot109, LjRoot170), HipMer performed better on single samples, while OPERA-MS was better on pooled samples (Supplementary Fig. 5), and other assemblers traded a higher genome fraction against more mismatches. Thus, coassembly could generally improve assembly for OPERA-MS and for short-read assemblers on low coverage genomes without expected strain diversity across samples. For HipMer, single-sample assembly might be preferable if coverage is sufficient and closely related strains are expected.

### Genome binning challenge

Genome binners group contigs or reads to recover genomes from metagenomes. We evaluated 95 results for 18 binner versions on short-read assemblies: 22 for the strain-madness GSAs, 17 for the strain-madness MEGAHIT assembly (MA), 19 for marine MA, 15 for marine GSA, 12 for plant-associated GSA and ten for the plant-associated MA (Supplementary Tables 9–15). In addition, seven results on the plant-associated hybrid assemblies were evaluated. Methods included well performing ones from the first CAMI challenge and popular software (Supplementary Table 2). While for GSA contigs the ground truth genome assignment is known, for the MA, we considered this to be the best matching genomes for a contig identified using MetaQUAST v.5.0.2. We assessed the average bin purity and genome completeness (and their summary using the F1-score), the number of high-quality genomes recovered, as well as the adjusted Rand index (ARI), using AMBER v.2.0.3 (ref. ^36^) (Methods). The ARI, together with the fraction of binned data, quantifies binning performance for the overall dataset.

**Fig. 2:**
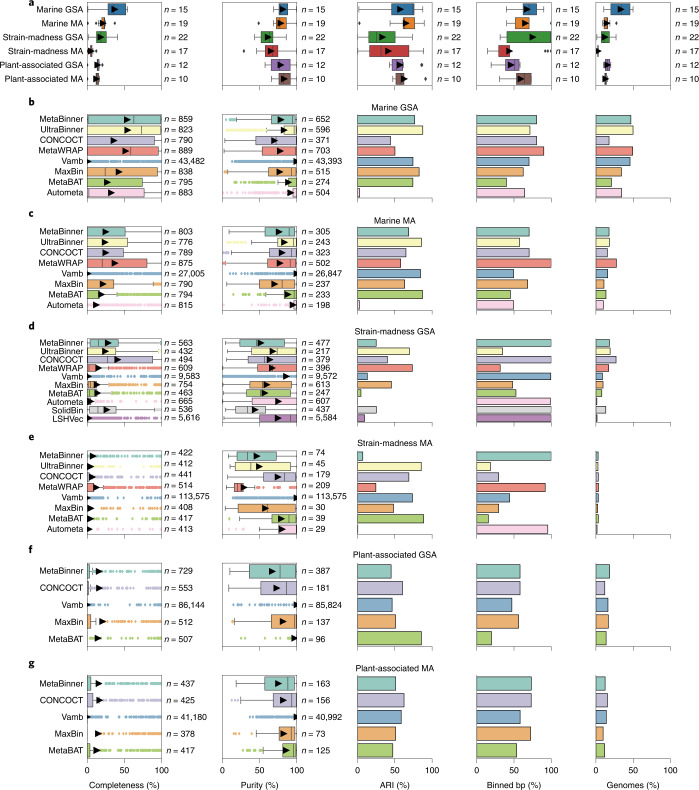
Performance of genome binners on short-read assemblies (GSA and MA, MEGAHIT) of the marine, strain-madness, and plant-associated data. **a**, Boxplots of average completeness, purity, ARI, percentage of binned bp and fraction of genomes recovered with moderate or higher quality (>50% completeness, <10% contamination) across methods from each dataset (Methods). Arrows indicate the average. **b**–**g**, Boxplots of completeness per genome and purity per bin, and bar charts of ARI, binned bp and moderate or higher quality genomes recovered, by method, for each dataset: marine GSA (**b**), marine MA (**c**), strain-madness GSA (**d**), strain-madness MA (**e**), plant-associated GSA (**f**) and plant-associated MA (**g**). The submission with the highest F1-score per method on a dataset is shown (Supplementary Tables 9–15). Boxes in boxplots indicate the interquartile range of *n* results, the center line the median and arrows the average. Whiskers extend to 1.5 × interquartile range or to the maximum and minimum if there is no outlier. Outliers are results represented as points outside 1.5 × interquartile range above the upper quartile and below the lower quartile. Source data

The performance of genome binners varied across metrics, software versions, datasets and assembly type (Fig. 2), while parameters affected performance mostly by less than 3%. For the marine GSA, average bin purity was 81.3 ± 2.3% and genome completeness was 36.9 ± 4.0% (Fig. 2a,b and Supplementary Table 9). For the marine MA, average bin purity (78.3 ± 2.6%) was similar, while average completeness was only 21.2 ± 1.6% (Fig. 2a,c and Supplementary Table 10), due to many short contigs with 1.5–2 kb, which most binners did not bin (Supplementary Fig. 6). For the strain-madness GSA, average purity and completeness decreased, by 20.1 to 61.2 ± 2.3% and by 18.7 to 18.2 ± 2.2%, respectively, relative to the marine GSA (Fig. 2a,d and Supplementary Table 11). While the average purity on the strain-madness MA (65.3 ± 4.0%) and GSA were similar, the average completeness dropped further to 5.2 ± 0.6%, again due to a larger fraction of unbinned short contigs (Fig. 2a,e and Supplementary Table 12). For the plant-associated GSA, purity was almost as high as for marine (78.2 ± 4.5%; Fig. 2a,f and Supplementary Table 13), but bin completeness decreased relative to other GSAs (13.9 ± 1.4%), due to poor recovery of low abundant, large, fungal genomes. Notably, the *Arabidposis thaliana* host genome (5.6x coverage) as well as fungi with more than eight times coverage were binned with much higher completeness and purity (Supplementary Fig. 7). Binning of the hybrid assembly further increased average purity to 85.1 ± 6.3%, while completeness remained similar (11.9 ± 2.1%, Supplementary Table 14). For the plant-associated MA, average purity (83 ± 3.3%) and completeness (12.4 ± 1.5%, Fig. 2a,g and Supplementary Table 15) were similar to the GSA.

To quantitatively assess binners across gold standard and real assemblies for the datasets, we ranked submissions (Supplementary Tables 16–19 and Supplementary Fig. 8) across metrics (Methods). For marine and strain-madness, CONCOCT^37^ and MetaBinner had the best trade-off performances for MAs, UltraBinner for GSAs and MetaBinner overall. CONCOCT also performed best on plant-associated assemblies (Table 1). UltraBinner had the best completeness on the marine GSA, CONCOCT on the strain-madness GSA and plant-associated MA, MetaWRAP on marine and strain-madness MAs and MaxBin^38^ on the plant-associated GSA. Vamb always had the best purity, while UltraBinner had the best ARI for the marine GSA, MetaWRAP for the strain-madness GSA and MetaBAT^39,40^ for MAs and plant-associated assemblies. MetaWRAP and MetaBinner assigned the most for the marine and plant-associated assemblies, respectively. Many methods assigned all strain-madness contigs, although with low ARI (Fig. 2b–g). UltraBinner recovered the most high-quality genomes from the marine GSA, MetaWRAP from the marine MA, CONCOCT from strain-madness assemblies and plant-associated GSA, and MetaBinner from the plant-associated GSA and hybrid assemblies (Fig. 2 and Supplementary Table 20). For plasmids and other high-copy circular elements, Vamb performed best, with an F1-score of 70.8%, 54.8% completeness and 100% purity, while the next best method, MetaWRAP, had an F1-score of 12.7% (Supplementary Table 21).

### Effect of strain diversity

For marine and strain-madness GSAs, unique strain binning was substantially better than for common strains (Supplementary Fig. 9 and Supplementary Tables 9 and 11). Differences were more pronounced on strain-madness, for which unique strain bin purity was particularly high (97.9 ± 0.4%). UltraBinner ranked best across metrics and four data partitions for unique genomes and overall, and CONCOCT for common strains (Supplementary Table 22). UltraBinner had the highest completeness on unique strains, while CONCOCT ranked best for common strains and across all partitions. Vamb always ranked first by purity, UltraBinner by ARI and MetaBinner by most assigned. Due to the dominance of unique strains in the marine and common strains in the strain-madness dataset, the best binners in the respective data and entire datasets were the same (Supplementary Tables 9 and 11) and performances similar for most metrics.

### Taxonomic binning challenge

Taxonomic binners group sequences into bins labeled with a taxonomic identifier. We evaluated 547 results for nine methods and versions: 75 for the marine, 405 for strain-madness and 67 for plant-associated data, on either reads or GSAs (Supplementary Tables 2). We assessed the average purity and completeness of bins and the accuracy per sample at different taxonomic ranks, using the National Center for Biotechnology Information (NCBI) taxonomy version provided to participants (Methods).

**Fig. 3:**
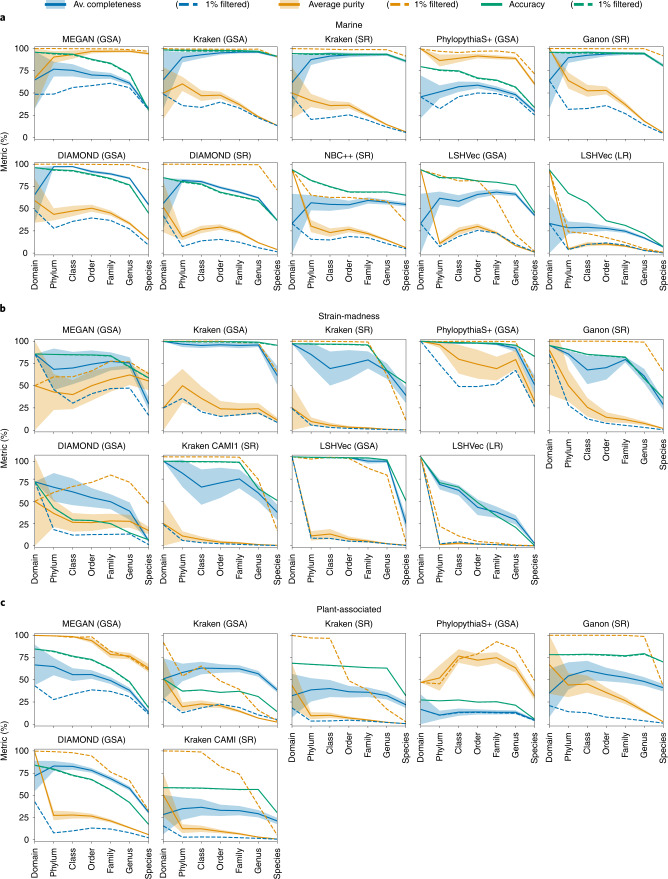
Taxonomic binning performance across ranks per dataset. **a**, Marine. **b**, Strain-madness. **c**, Plant-associated. Metrics were computed over unfiltered (solid lines) and 1%-filtered (that is, without the 1% smallest bins in bp, dashed lines) predicted bins of short reads (SR), long reads (LR) and contigs of the GSA. Shaded bands show the standard error across bins. Source data

On the marine data, average taxon bin completeness across ranks was 63%, average purity 40.3% and accuracy per sample bp 74.9% (Fig. 3a and Supplementary Table 23). On the strain-madness data, accuracy was similar (76.9%, Fig. 3b and Supplementary Table 24), while completeness was around 10% higher and purity lower by that much. On the plant-associated data, purity was between those of the first two datasets (35%), but completeness and accuracy were lower (44.2 and 50.8%, respectively; Fig. 3c and Supplementary Table 25). For all datasets, performances declined at lower taxonomic ranks, most notably from genus to species rank by 22.2% in completeness, 9.7% in purity and 18.5% in accuracy, on average.

Across datasets, MEGAN on contigs ranked first across metrics and ranks (Supplementary Table 26), closely followed by Kraken v.2.0.8 beta on contigs and Ganon on short reads. Kraken on contigs was best for genus and species, and on marine data across metrics and in completeness and accuracy (89.4 and 96.9%, Supplementary Tables 23 and 27 and Supplementary Fig. 10). Due to the presence of public genomes, Kraken’s completeness on marine data was much higher than in the first CAMI challenge, particularly at species and genus rank (average of 84.6 and 91.5%, respectively, versus 50 and 5%), while purity remained similar. MEGAN on contigs ranked highest for taxon bin purity on the marine and plant-associated data (90.7 and 87.1%, Supplementary Tables 23, 25, 27 and 28). PhyloPythiaS+ ranked best for the strain-madness data across metrics, as well as in completeness (90.5%) and purity (75.8%) across ranks (Supplementary Tables 24 and 29). DIAMOND on contigs ranked best for completeness (67.6%) and Ganon on short reads for accuracy (77.1%) on the plant-associated data.

Filtering the 1% smallest predicted bins per taxonomic level is a popular postprocessing approach. Across datasets, filtering increased average purity to above 71% and reduced completeness, to roughly 24% on marine and strain-madness and 13.4% on plant-associated data (Supplementary Tables 23–25). Accuracy was not much affected, as large bins contribute more to this metric. Kraken on contigs still ranked first in filtered accuracy and MEGAN across all filtered metrics (Supplementary Table 26). MEGAN on contigs and Ganon on short reads profited the most from filtering, ranking first in filtered completeness and purity, respectively, across all datasets and taxonomic levels.

### Taxonomic binning of divergent genomes

To investigate the effect of increasing divergence between query and reference sequences for taxonomic binners, we categorized genomes by their distances to public genomes (Supplementary Fig. 11 and Supplementary Tables 30 and 31). Sequences of known marine strains were assigned particularly well at species rank by Kraken (accuracy, completeness and filtered purity above 93%) and MEGAN (91% purity, 33% completeness and accuracy). Kraken also best classified new strain sequences at species level, although with less completeness and accuracy for the marine data (68 and 80%, respectively). It also had the best accuracy and completeness across ranks, but low unfiltered purity. For the strain-madness data, PhyloPythiaS+ performed similarly well up to genus level and best assigned new species at genus level (93% accuracy and completeness, and 75% filtered purity). Only DIAMOND correctly classified viral contigs, although with low purity (50%), completeness and accuracy (both 3%).

### Taxonomic profiling challenge

Taxonomic profilers quantify the presence and relative taxon abundances of microbial communities from metagenome samples. This is different from taxonomic sequence classification, which assigns taxon labels to individual sequences and results in taxon-specific sequence bins (and sequence abundance profiles)^41^. We evaluated 4,195 profiling results (292 marine, 2,603 strain-madness and 1,300 plant-associated datasets), from 22 method versions (Supplementary Table 2) with most results for short-read samples, and a few for long-read samples, assemblies or averages across samples. Performance was evaluated with OPAL v.1.0.10 (ref. ^42^) (Methods). The quality of predicted taxon profiles was determined based on completeness and purity of identified taxa, relative to the underlying ground truth, for individual ranks, while taxon abundance estimates were assessed using the L1 norm for individual ranks and the weighted UniFrac error across ranks. Accuracy of alpha diversity estimates was measured using the Shannon equitability index (Methods). Overall, mOTUs v.2.5.1 and MetaPhlAn v.2.9.22 ranked best across taxonomic ranks and metrics on the marine and plant-associated datasets, and mOTUs v.cami1 and MetaPhlAn v.2.9.22 on the strain-madness dataset (Table 1, Supplementary Tables 33, 35 and 37 and Supplementary Fig. 12).

### Taxon identification

**Fig. 4:**
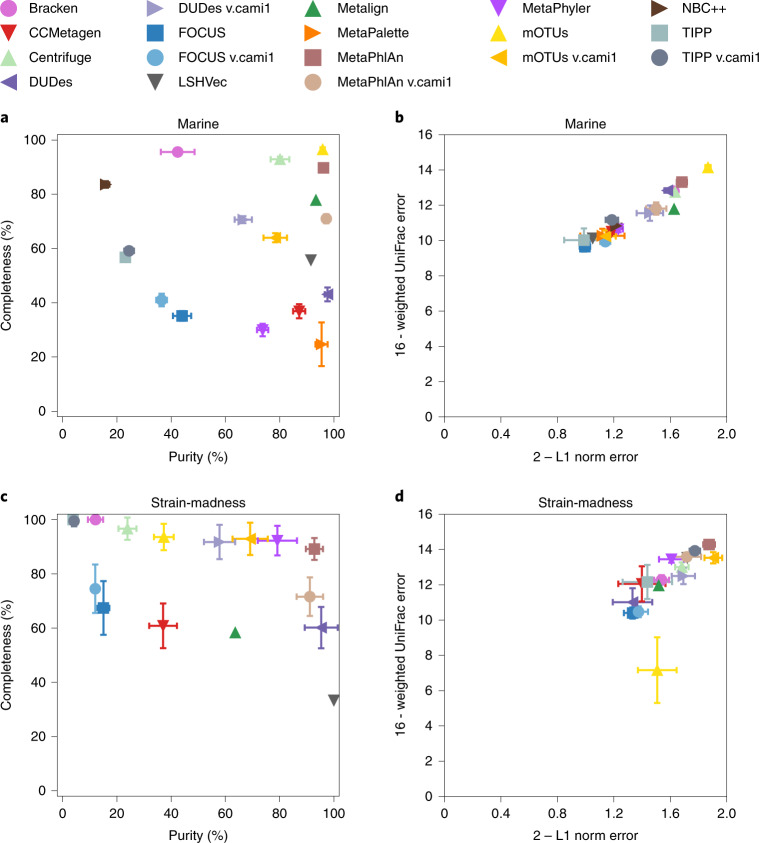
Taxonomic profiling results for the marine and strain-madness datasets at genus level. **a**,**b**, Marine datasets. **c**,**d**, Strain-madness datasets. Results are shown for the overall best ranked submission per software version (Supplementary Tables 33 and 35, and Supplementary Fig. 12). **a**,**c**, Purity versus completeness. **b**,**d**, Upper bound of L1 norm (2) minus actual L1 norm versus upper bound of weighted UniFrac error (16) minus actual weighted UniFrac error. Symbols indicate the mean over ten marine and 100 strain-madness samples, respectively, and error bars the standard deviation. Metrics were determined using OPAL with default settings. Source data

Methods performed well until genus rank (marine average purity 70.4%, strain-madness 52.1%, plant-associated 62.9%; marine average completeness 63.3%, strain-madness 80.5%, plant-associated 42.1%; Fig. 4a,c, Supplementary Fig. 13 and Supplementary Tables 32, 34 and 36), with a substantial drop at species level. mOTUs v.2.5.1 (ref. ^43^) had completeness and purity above 80% at genus and species ranks on marine data, and Centrifuge v.1.0.4 beta (ref. ^44^) and MetaPhlAn v.2.9.22 (refs. ^45,46^) at genus rank (Fig. 4a). Bracken^47^ and NBC++ (ref. ^48^) had completeness above 80% at either rank, and CCMetagen^49^, DUDes v.0.08 (ref. ^50^), LSHVec v.gsa^51^, Metalign^52^, MetaPalette^53^ and MetaPhlAn v.cami1 more than 80% purity. Filtering the rarest (1%) predicted taxa per rank decreased completeness by roughly 22%, while increasing precision by roughly 11%.

On strain-madness data at genus rank, MetaPhlAn v.2.9.22 (89.2% completeness, 92.8% purity), MetaPhyler v.1.25 (ref. ^54^) (92.3% completeness, 79.2% purity) and mOTUs v.cami1 (92.9% completeness, 69.1% purity) performed best, but no method excelled at species rank. DUDes v.0.08 and LSHVec v.gsa had high purity, while Centrifuge v.1.0.4 beta, DUDes v.cami1, TIPP v.4.3.10 (ref. ^55^) and TIPP v.cami1 high completeness.

On plant-associated data at genus rank, sourmash_gather v.3.3.2_k31_sr (ref. ^56^) was best overall (53.3% completeness, 89.5% purity). Sourmash_gather v.3.3.2_k31 on PacBio reads and MetaPhlAn v.3.0.7 had the highest purity for genus (98.5%, 95.5%) and species ranks (64.4%, 68.8%) and sourmash_gather v.3.3.2_k21_sr the highest completeness (genus 61.9%, species 53.8%).

### Relative abundances

Abundances across ranks and submissions were on average predicted better for strain-madness than marine data, which has less complexity above strain level, with the L1 norm improving from 0.44 to 0.3, and average weighted UniFrac error from 4.65 to 3.79 (Supplementary Tables 32, 34 and 36). These weighted UniFrac values are substantially higher than for biological replicates (0.22, Methods). Abundance predictions were not as good on the plant-associated data and averaged 0.57 in L1 norm and 5.16 in weighted UniFrac. On the marine data, mOTUs v.2.5.1 had the lowest L1 norm at almost all levels with 0.12 on average, 0.13 at genus and 0.34 at species level, respectively. It was followed by MetaPhlAn v.2.9.22 (average 0.22, 0.32 genus, 0.39 species). Both methods also had the lowest weighted UniFrac error, followed by DUDEs v.0.08. On the strain-madness data, mOTUs v.cami1 performed best in L1 norm across ranks (0.05 average), and also at genus and species with 0.1 and 0.15, followed by MetaPhlAn v.2.9.22 (0.09 average, 0.12 genus, 0.23 species). The last also had the lowest weighted UniFrac error, followed by TIPP v.cami1 and mOTUs v.2.0.1. On the plant-associated data, Bracken v.2.6 had the lowest L1 norm across ranks with 0.36 on average, and at genus with 0.34. Sourmash_gather v.3.3.2_k31’ on short reads had the lowest (0.55) at species. Both methods also had the lowest UniFrac error on this dataset. Several methods accurately reconstructed the alpha diversity of samples using the Shannon equitability; best (0.03 or less absolute difference to gold standards) across ranks on marine data were: mOTUs v.2.5.1, DUDes v.0.08 and v.cami1 and MetaPhlAn v.2.9.22 and v.cami1; on strain-madness data: DUDes v.cami1, mOTUs v.cami1 and MetaPhlAn v.2.9.22. On the plant-associated data, mOTU v.cami1 and Bracken v.2.6 performed best with this metric (0.08 and 0.09).

### Difficult and easy taxa

For all methods, viruses, plasmids and Archaea were difficult to detect (Supplementary Fig. 14 and Supplementary Table 38) in the marine data. While many Archaeal taxa were detected by several methods, others, such as Candidatus Nanohaloarchaeota, were not detected at all. Only Bracken and Metalign detected viruses. In contrast, bacterial taxa in the Terrabacteria group and the phyla of Bacteroidetes and Proteobacteria were always correctly detected. Based on taxon-wise precision and recall for submissions, methods using similar information tended to cluster (Supplementary Fig. 15).

### Clinical pathogen prediction: a concept challenge

Clinical pathogen diagnostics from metagenomics data is a highly relevant translational problem requiring computational processing^57^. To raise awareness, we offered a concept challenge (Methods): a short-read metagenome dataset of a blood sample from a patient with hemorrhagic fever was provided for participants to identify pathogens and to indicate those likely to cause the symptoms described in a case report. Ten manually curated, hence not reproducible results were received (Supplementary Table 39). The number of identified taxa per result varied considerably (Supplementary Fig. 16). Three submissions correctly identified the causal pathogen, Crimean–Congo hemorrhagic fever orthonairovirus (CCHFV), using the taxonomic profilers MetaPhlAn v.2.2, Bracken v.2.5 and CCMetagen v.1.1.3 (ref. ^49^). Another submission using Bracken v.2.2 correctly identified orthonairovirus, but not as the causal pathogen.

### Computational requirements

**Fig. 5:**
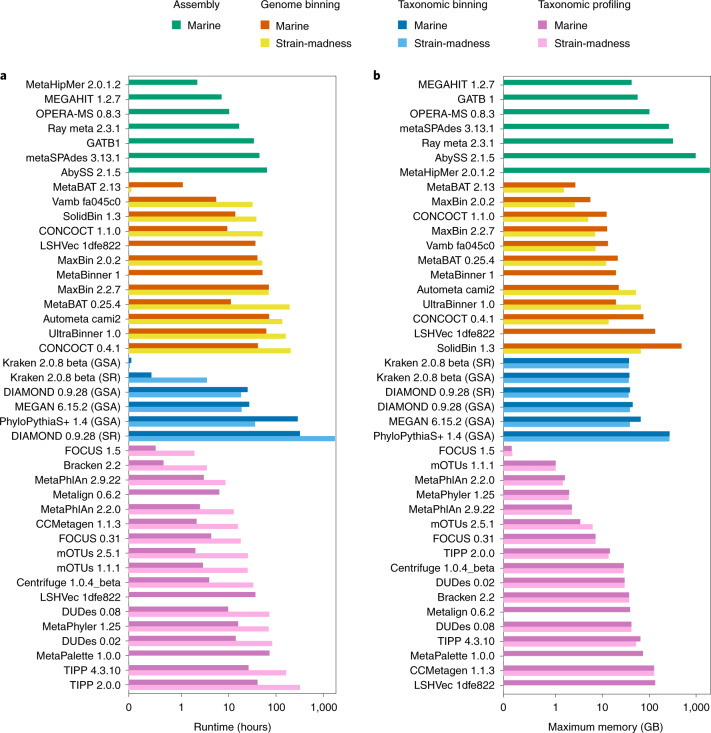
Computational requirements of software from all categories. **a**, Runtime. **b**, Maximum memory usage. Results are reported for the marine and strain-madness read data or GSAs (Supplementary Table 40). The *x* axes are log scaled and the numbers given are the software version numbers. Source data

We measured the runtimes and memory usage for submitted methods across the marine and strain-madness data (Fig. 5, Supplementary Table 40 and Methods). Efficient methods capable of processing the entire datasets within minutes to a few hours were available in every method category, including some top ranked techniques with other metrics. Substantial differences were seen within categories and even between versions, ranging from methods executable on standard desktop machines to those requiring extensive hardware and heavy parallelization. MetaHipMer was the fastest assembler and required 2.1 h to process marine short reads, 3.3× less than the second fastest assembler, MEGAHIT. However, MetaHipMer used the most memory (1,961 gigabytes (GB)). MEGAHIT used the least memory (42 GB), followed by GATB (56.6 GB). On marine assemblies, genome binners on average required roughly three times less time than for the smaller strain-madness assemblies (29.2 versus 86.1 h), but used almost 4× more memory (69.9 versus 18.5 GB). MetaBAT v.2.13.33 was the fastest (1.07 and 0.05 h) and most memory efficient binner (maximum memory usage 2.66 and 1.5 GB) on both datasets. It was roughly 5× and 635× faster than the second fastest method, Vamb v.fa045c0, roughly 6× faster than LSHVec v.1dfe822 on marine and 765× faster than SolidBin v.1.3 on strain-madness data; roughly twice and 5× more memory efficient than the next ranking MaxBin v.2.0.2 and CONCOCT v.1.1.0 on marine data, respectively. Both MetaBAT and CONCOCT were substantially (roughly 11× and 4×) faster than their CAMI 1 versions. Like genome binners, taxonomic binners ran longer on the marine than the strain-madness assemblies, for example PhyloPythiaS+ with 287.3 versus 36 h, respectively, but had a similar or slightly higher memory usage. On the marine read data, taxon profilers, however, were almost 4× faster on average (16.1 versus 60.8 h) than on the ten times larger strain-madness read dataset, but used more memory (38.1 versus 25 GB). The fastest and most memory efficient taxonomic binner was Kraken, requiring only 0.05 and 0.02 h, respectively, and roughly 37 GB memory on both datasets, for reads or contigs. It was followed by DIAMOND, which ran roughly 500× and 910× as long on the marine and strain-madness GSAs, respectively. FOCUS v.1.5 (ref. ^58^) and Bracken v.2.2 were the fastest profilers on the marine (0.51, 0.66 h, respectively) and strain-madness (1.89, 3.45 h) data. FOCUS v.1.5 also required the least memory (0.16 GB for marine, 0.17 GB for strain-madness), followed by mOTUs v.1.1.1 and MetaPhlAn v.2.2.0.

## Discussion

Assessing metagenomic analysis software thoroughly, comprehensively and with little bias is key for optimizing data processing strategies and tackling open challenges in the field. In its second round, CAMI offered a diverse set of benchmarking challenges across a comprehensive data collection reflecting recent technical developments. Overall, we analyzed 5,002 results of 76 program versions with different parameter settings across 131 long- and short-read metagenome samples from four datasets (marine, plant-associated, strain-madness, clinical pathogen challenge). This effort increased the number of results 22× and the number of benchmarked software versions 3× relative to the first challenge, delivering extensive new insights into software performances across a range of conditions. By systematically assessing runtime and memory requirements, we added two more key performance dimensions to the benchmark, which are important to consider given the ever-increasing dataset sizes.

In comparison to software assessed in the first challenges, assembler performances rose by up to 30%. Still, in the presence of closely related strains, assembly contiguity, genome fractions and strain recall decreased, suggesting that most assemblers, sometimes intentionally^19,26^, did not resolve strain variation, resulting in more fragmented, less strain-specific assemblies. In addition, genome coverage, parameter settings and data preprocessing impacted assembly quality, while performances were similar across software versions. Most submitted metagenome assemblies used only short reads, and long and hybrid assemblies had no higher overall quality. Hybrid assemblies, however, were better for difficult to assemble regions, such as the 16S rRNA gene, recovering more complete genes than most short-read submissions. Hybrid assemblers were also less affected by closely related strains in pooled samples, suggesting that long reads help to distinguish strains.

In comparison to the first CAMI challenges, ensemble binners presented a development showing substantial improvements across metrics compared to most individual methods. Overall, genome binners demonstrated variable performances across metrics and dataset types, with strain diversity and lower assembly quality presenting challenges that substantially reduced performances, even for the large sample number of the strain-madness dataset. For the plant host and 55 fungal genomes with sufficient coverage in the plant-associated data, high-quality bins were also obtained.

For taxonomic binners and profilers, highly performant and computationally efficient software was available, performing well across a range of conditions and metrics. Particularly profilers have matured since the first challenges, with less variance in top performers across taxon identification, abundance and diversity estimates. Performance was high for genus rank and above, with a substantial drop for bacterial species. As the second challenge data include high-quality public genomes, the data are less divergent from publicly available data than for the first challenges, on which method performances had already declined going from family to genus rank. It was also low for Archaea and viruses, suggesting a need for developers to extend their reference sequence collections and model development. Another encouraging result is that in the clinical pathogen challenge, several submissions identified the causal pathogen. However, due to manual curation, none was reproducible, indicating that these methods still require improvements, as well as assessment on large data collections. Although there is great potential of clinical metagenomics for pathogen diagnostics and characterization^57^, multiple challenges still prevent its application in routine diagnostics^59^.

In its second challenge, CAMI identified key advances for common metagenomics software categories as well as current challenges. As the state-of-the-art in methods and data generation progresses, it will be important to continuously re-evaluate these questions. In addition, computational methods for other microbiome data modalities^6^ and multi-omics data integration could be jointly assessed. Most importantly, CAMI is a community-driven effort and we encourage everyone interested in benchmarking in microbiome research to join us.

## Methods

### Community involvement

We gathered community input on the nature and principles of implementing benchmarking challenges and datasets in public workshops and hackathons (https://www.microbiome-cosi.org/cami/participate/schedule). The most relevant metrics for performance evaluation and data interpretation were discussed in a public workshop with challenge participants and developers of evaluation software where first challenge results were presented in an anonymized manner. Computational support for challenge participants was provided by the de.NBI cloud.

### Standardization and reproducibility

To ensure reproducibility and assess computational behavior (runtimes and memory consumption) of the software used to create challenge submissions, we reproduced and reassessed the results according to submission specifications (Supplementary Table 2, https://data.cami-challenge.org/). For metagenome assemblers, computational requirements were assessed on a machine with Intel Xeon Processor (2.6 GHz) virtualized to 56 cores (50 cores used) and 2,755 GB of main memory and, for binners and profilers, on a machine with an Intel Xeon E5-4650 v4 CPU (virtualized to 16 CPU cores, one thread per core) and 512 GB of main memory. Methods were executed one at a time and exclusively on each hardware. We also updated Docker BioContainers implementing a range of commonly used performance metrics to include all metrics used in this evaluation (MetaQUAST^17^: https://quay.io/repository/biocontainers/quast, AMBER^36^ and https://quay.io/repository/biocontainers/cami-amber, OPAL^42^: https://quay.io/repository/biocontainers/cami-opal).

### Genome sequencing and assembly

Illumina paired-end read data of 796 newly sequenced genomes, of which 224 stem from an *Arabidopsis thaliana* root environment, 176 from a marine environment^60^, 384 clinical *Streptococcus pneumoniae* strains and 12 strains from a murine gut environment, were assembled using a pipeline with the SPAdes^61^ metagenome assembler (v.3.12). We removed contigs smaller than 1 kb, and genome assemblies with a contamination of 5% or more and completeness of 90% or less, as determined with CheckM^62^ v.1.011. Newly assembled and database genomes were taxonomically classified with CAMITAX^63^ and used as input for microbial community and metagenome data simulation with CAMISIM^16^, based on the from_profile mode for the marine and plant-associated dataset and the de novo mode for the strain-madness datasets. All scripts and parameters for these steps are provided in the Supplementary Material and on GitHub (https://github.com/CAMI-challenge/second_challenge_evaluation/tree/master/scripts/data_generation).

For the plasmid dataset, inlet wastewater from a wastewater treatment plant on Zealand, Denmark was used to generate a plasmid sample similar to ref. ^64^. Sequencing was performed on a NextSeq 500 on Nextera sequencing libraries (Illumina). A bioinformatic workflow described in ref. ^65^ was used to identify complete circular plasmids above 1 kb in size in the dataset.

### Challenge datasets

For the challenges, participants were provided with long- and short-read sequences for two metagenome datasets representing a marine and a plant-associated environment, respectively, and for a ‘strain-madness’ dataset with very high strain diversity. Furthermore, a short-read clinical metagenomic dataset from a critically ill patient was provided.

The ten-sample 100 GB marine dataset was created with CAMISIM from BIOM profiles of a deep-sea environment, using 155 newly sequenced marine isolate genomes from this environment and 622 genomes with matching taxonomic provenance from MarRef^66^, a manually curated database with completely sequenced marine genomes. Of these genomes, 303 (39%)—204 database genomes (31.9%) and 99 new genomes (72.3%)—have a closely related strain present, with an ANI of 95% or more. Additionally, 200 newly sequenced circular elements including plasmids and viruses were added. For each sample, 5 gigabase (Gb) of paired-end short Illumina and long Pacific Biosciences reads were created (Supplementary Text).

The 100-sample 400 GB strain-madness dataset includes 408 newly sequenced genomes, of which 97% (395) had a closely related strain. For each sample, 2 Gb of paired-end short and long-read sequences were generated with CAMISIM, respectively, using the same parameters and error profiles as in CAMI 1 (ref. ^4^) (Supplementary Text).

The 21-sample 315 GB plant-associated dataset includes 894 genomes. Of these, 224 are from the proGenomes^67^ terrestrial representative genomes, 216 are newly sequenced genomes from an *A. thaliana* root rhizosphere, 55 are fungal genomes associated with the rhizosphere^68^, 398 are plasmids or circular elements and one *A. thaliana* genome. Of these genomes, 15.3% (137) have at least one closely related genome present. For each sample, 5 Gb of paired-end short-read sequences, as well as 2 × 5 Gb long-read sequences mimicking Pacific Biosciences and Oxford Nanopore sequencing data, respectively, were generated. Note that 90% of metagenome sequence data originate from bacterial genomes, 9% are fungal genome sequences and 1% is from *A. thaliana*. To evaluate the assembly quality of single-sample versus cross-assembly strategies, 23 new genomes from eight clusters of closely related genomes were selected and added to the dataset in certain samples with predetermined abundances. For all three datasets, we generated gold standards for every metagenome sample individually and for the pooled samples, which included assemblies for short, long and hybrid reads, genome bin and taxon bin assignments and taxonomic profiles.

Finally, a 688-MB paired-end MiSeq metagenomic sequencing dataset of a blood sample from a patient with hemorrhagic fever was provided. Previous analysis of the sample had revealed sequences matching the genome of CCHFV (NCBI taxid 1980519), and the presence of the viral genome was subsequently confirmed via PCR (with a cycle threshold value of 27.4). The causative nature of CCHFV could not be clinically proved due to the provenance of the original sample and CCHFV has previously been shown to cause subclinical infections^69^. However, no evidence of other pathogens that could cause hemorrhagic fever was found in the sample, making causality of CCHFV the most plausible explanation of the symptoms. To create a realistic dataset and case for the challenge while protecting the identity of the patient, the clinical case description was derived from the true anamnesis and modified in ways consistent with the causative agent. Additionally, reads mapping to the human genome were replaced by sequences from the same genomic regions randomly drawn from the 1,000 genomes dataset^70^. Challenge participants were asked to identify the causal pathogen as well as all other pathogens present in the sample.

### Challenge organization

The second round of CAMI challenges assessed software for metagenome assembly, genome binning, taxonomic binning, taxonomic profiling and diagnostic pathogen prediction. As before, two metagenome ‘practice’ benchmark datasets were created from public genomes and provided together with the ground truth before the challenges, to enable contest participants to familiarize themselves with data types and formats. These included a 49-sample dataset modeled from Human Microbiome data^12,35^ and a 64-sample dataset modeled in taxonomic composition from mouse gut samples^71,72^, with 5 Gb long (Pacific Biosciences, variable length with a mean of 3,000 bp) and 5 Gb short (Illumina HiSeq2000, 150 bp) paired-end read sequences, respectively. Read profiles (read length and error rates) were created from sequencing runs on the MBARC-26 dataset^73^. Reference data collections with NCBI RefSeq, nr/nt and taxonomy from 8 January 2019 were provided to participants, for use with reference-based methods in the challenges. To reduce differences in taxonomy due to eventual use of precompiled reference databases by taxonomic binners, NCBI’s merged.dmp file was used to map synonymous taxa during assessments.

The second challenge started on 16 January 2019 (https://www.microbiome-cosi.org/cami/cami/cami2). Participants registered for download of the challenge datasets, with 332 teams registering from that time until January 2021. For reproducibility, participants could submit a Docker container containing the complete workflow, a bioconda script or a software repository with detailed installation instructions specifying all parameter settings and reference databases used. Assembly results could be submitted for short-read data, long-read data or both data types combined. For methods incapable of submitting a cross-sample assembly for the entire dataset, a cross-sample assembly for the first ten samples of a dataset could be submitted. Participants could also submit single-sample assemblies for each of the first five samples of a dataset. Specification of the performance criteria for strain-aware assembly can be found in the Supplementary Material. The assembly challenge closed on 17 May 2019. Immediately afterward, gold standard and MEGAHIT^19^ assemblies were provided for both datasets. The GSAs include all sequences of the reference genomes and circular elements covered by one short read in the combined metagenome datasets. Analysis of GSA binnings allowed us to assess binning performances independently of assembly quality. We assessed the contributions of assembly quality by comparing with the binning results on MEGAHIT assemblies. Profiling results were submitted for all individual samples and for the entire datasets, respectively. Binning results included genome or taxon bin assignments for analyzed reads or contigs of the provided assemblies for every sample of a dataset. Results for the pathogen detection challenge included predictions of all pathogens and a causal pathogen responsible for the symptoms outlined in a clinical case description provided together with the clinical metagenome dataset. The CAMI II challenges ended on 25 October 2019. Subsequently, another round of challenges (‘CAMI II b’) on plant-associated data was offered starting on 14 February 2020. This closed on 29 September 2020 for assembly submissions and on 31 January 2021 for genome and taxonomic binning, as well as profiling.

Altogether 5,002 submissions of 76 programs were received for the four challenge datasets, from 30 external teams and CAMI developers (Supplementary Table 2). All genome data used for generation of the benchmark datasets as well as their metadata were kept confidential during the challenge and released afterward (10.4126/FRL01-006421672). To support an unbiased assessment, program submissions were represented with anonymous names in the portal (known only to submitters) and a second set of anonymous names for evaluation and discussion in the evaluation workshop, such that identities were unknown to all except for the data analysis team (F.Meyer, Z.-L.D., A.F., A.S.) and program identities revealed only after a first consensus was reached.

### Evaluation metrics

In the following, we briefly outline the metrics used to evaluate the four software categories. For details, the reader is also referred to refs. ^36,42^.

#### Assemblies

Assemblies were evaluated with metaQUAST v.5.1.0rc using the --unique-mapping flag. This flag allows every contig to be mapped at only a single reference genome position. We focused on commonly used assembly metrics such as genome fraction, mismatches per 100 kb, duplication ratio, NGA50 and the number of misassemblies. The genome fraction specifies the percentage of reference bases covered by assembled contigs after similarity-based mapping. Mismatches per 100 kb specify the number of mismatched bases in the contig-reference alignment. The duplication ratio is defined as the total number of aligned bases of the assembly divided by the total number of aligned bases of the reference genome. NGA50 is a metric for measuring the contiguity of an assembly. For each reference genome, all aligned contigs are sorted by size. The NGA50 for that genome is defined as the length of the contig cumulatively surpassing 50% genome fraction. If a genome is not covered to 50%, NGA50 is undefined. Since we report the average NGA50 over all genomes, it was set to 0 for genomes with less than 50% genome fraction. Finally, the number of misassemblies describes the number of contigs that contain a gap of more than 1 kb, contain inserts of more than 1 kb or align to two or more different genomes. In addition to these metrics, similar to ref. ^18^ we determined the strain recall and strain precision to quantify the presence of high-quality, strain-resolved assemblies. Strain recall is defined as the fraction of high-quality (more than 90% genome fraction and less than a specific number of mismatches per 100 kb) genome assemblies recovered for all ground truth genomes. Strain precision specifies the fraction of low mismatch and high genome fraction (more than 90%) assemblies among all high genome fraction assemblies. For the strain-madness dataset, the required genome fraction was set to 75% and allowed mismatches to <0.5%, because of the generally lower assembly quality.

For the genome binning, for every predicted genome bin *b*, the true positives TP_*b*_ are the number of base pairs of the most abundant genome *g* in *b*, the false positives FP_*b*_ are the number of base pairs in *b* belonging to genomes other than *g* and the false negatives FN_*b*_ are the number of base pairs belonging to *g* that are not in *b*.

Purity is defined for each predicted genome bin *b* as:

$${\mathrm{purity}}_b = \frac{{{\mathrm{TP}}_b}}{{{\mathrm{TP}}_b + {\mathrm{FP}}_b}}.$$

The average purity is a simple average of the purity of bins *b* in the set of all predicted genome bins *B*, that is:

$${\mathrm{average}}\,{\mathrm{purity}} = \frac{{\mathop {\sum}\nolimits_{b \in B} {{\mathrm{purity}}_b} }}{{\left| B \right|}}.$$

Completeness is defined for each genome *g* based on its mapping to a genome bin *b* that it is most abundant in, as:

$${\mathrm{completeness}}_{gb} = \frac{{{\mathrm{TP}}_{gb}}}{{{\mathrm{TP}}_{gb} + {\mathrm{FN}}_{gb}}}.$$

The average completeness is defined over all genomes in the sample, including those that are the most abundant in none of the predicted genome bins. Let *X* be the set of such genomes. The average completeness is then defined as:

$${\mathrm{average}}\,{\mathrm{completeness}} = \frac{{\mathop {\sum}\nolimits_{b \in B} {{\mathrm{completeness}}_{gb}} }}{{\left| B \right| + \left| X \right|}}.$$

As another metric, we consider the number of predicted genome bins that fulfill specific quality criteria. Bins with >50% completeness and <10% contamination are denoted as ‘moderate or higher’ quality bins and bins with completeness >90% and contamination <5% as high-quality genome bins, similar to CheckM^62^.

The ARI is defined as in ref. ^36^. The Rand index compares two clusterings of the same set of items. Assuming the items are base pairs of different sequences, base pairs belonging to the same genome that were binned together in the same genome bin are considered true positives, and base pairs belonging to different genomes that were put into different genome bins are considered true negatives. The Rand index is the sum of true positives and negatives divided by the total number of base pairs. The ARI takes into account that the Rand index can be above 0 by chance, normalized such that the result ranges between 1 (best), representing a perfect match of clusterings and close to 0 (worst, see ref. ^36^ for a complete definition) for a match no better than chance. As binning methods may leave a portion of the data unbinned, but the ARI is not suitable for datasets that are only partially assigned, it is computed for the binned portion only and interpreted together with the percentage of binned base pairs of a dataset.

For taxonomic binning, metrics are calculated for each of the major taxonomic ranks, from superkingdom or domain to species. Purity and completeness for each taxonomic bin *b* (that is, group of sequences and base pairs therein assigned to the same taxon) are computed by setting TP_*b*_ to the number of base pairs of the true taxon *t* assigned to *b*, FP_*b*_ the number of base pairs assigned to *b* belonging to other taxa and FN_*b*_ the number of base pairs of *t* not assigned to *b*. The average purity at a certain taxonomic rank is a simple average of the purity of all predicted taxon bins at that taxonomic rank.

The average completeness at a certain taxonomic rank is the sum of the completeness over all predicted taxon bins divided by the number of taxa, GS, in the gold standard at that taxonomic rank. That is:

$${\mathrm{average}}\,{\mathrm{completeness}} = \frac{{\mathop {\sum}\nolimits_{b \in B} {{\mathrm{completeness}}_b} }}{{\left| {{\mathrm{GS}}} \right|}}.$$

The accuracy at a certain taxonomic rank is defined as:

$${\mathrm{accuracy}} = \frac{{\mathop {\sum}\nolimits_{b \in B} {{\mathrm{TP}}_b} }}{n},$$

where *B* is the set of predicted taxon bins at that taxonomic rank and *n* is the total number of base pairs in GS for that taxonomic rank.

Average purity, completeness and accuracy are also computed for a filtered subset *B*_*f*_ of *B* of each taxonomic rank, without the 1% smallest bins, and are denoted below average purity_*f*_, ${\mathrm{average}}\,{\mathrm{completeness}}_f$ and accuracy_*f*_. *B*_*f*_ is obtained by sorting all bins in *B* by increasing size in base pairs and filtering out the first bins whose cumulative size sum is smaller or equal to 1% of summed size of all bins in *B*. These metrics are then computed as:

$${\mathrm{average}}\,{\mathrm{purity}}_f = \frac{{\mathop {\sum}\nolimits_{b \in B_f} {{\mathrm{purity}}_b} }}{{\left| {B_f} \right|}},$$

$${\mathrm{average}}\,{\mathrm{completeness}}_f = \frac{{\mathop {\sum}\nolimits_{b \in B_f} {{\mathrm{completeness}}_b} }}{{\left| {{\mathrm{GS}}} \right|}},$$

$${\mathrm{accuracy}}_f = \frac{{\mathop {\sum}\nolimits_{b \in B_f} {{\mathrm{TP}}_b} }}{n}.$$

For taxonomic profiling, we determined purity and completeness in taxon identification, L1 norm and weighted UniFrac^74^ as abundance metrics, and alpha diversity estimates using the Shannon equitability index, as outlined below.

The purity and completeness for a taxonomic profile measure a method’s ability to determine the presence and absence of taxa in a sample, at a certain taxonomic rank, without considering their relative abundances. Let the true positives, TP, and false positives, FP, be the number of correctly and incorrectly detected taxa, that is, taxa present or absent in the gold standard profile, respectively, for a certain sample and rank. Further, let the false negatives, FN, be the number of taxa that are in the gold standard profile but a method failed to detect. Purity, completeness and F1-score are then defined as above.

The L1 norm error, Bray–Curtis distance and weighted UniFrac error measure a method’s ability to determine the relative abundances of taxa in a sample. Except for the UniFrac metric (which is rank independent), these are defined at each taxonomic rank. Let *x*_*t*_ and $x_t^ \ast$ be the true and predicted relative abundances of taxon *t* in a sample, respectively. The L1 norm gives the total error between *x*_*t*_ and $x_t^ \ast$ in a sample, for all true and predicted *t* at a certain rank and ranges between 0 and 2. It is determined as:

$$L1\,{\mathrm{norm}}\,{\mathrm{error}} = \mathop {\sum}\nolimits_t {\left| {x_t - x_t^ \ast } \right|}$$

The Bray–Curtis distance is the L1 norm error divided by the sum of all abundances *x*_*t*_ and $x_t^ \ast$ at the respective rank, that is:

$${\mathrm{Bray}}-{\mathrm{Curtis}}\,{\mathrm{distance}} = \frac{{\mathop {\sum}\nolimits_t {\left| {x_t - x_t^ \ast } \right|} }}{{\mathop {\sum}\nolimits_t {x_t + x_t^ \ast } }}$$

The Bray–Curtis distance ranges between 0 and 1. As the gold standards usually contain abundances for 100% of the data, it is equal to half of the L1 norm error if the profiler made predictions also for 100% of the data, and higher otherwise.

The weighted UniFrac metric uses differences between predicted and actual abundances weighted by distance in the taxonomic tree. It ranges between 0 (best) and 16 (worst). The value of ‘16’ is present due to the fact that the NCBI taxonomy has eight major taxonomic ranks (kingdom, phylum, class and so on). As such, when using unit branch lengths, the worst possible UniFrac value is 16: the case when one sample contains 100% of its abundance in a different kingdom than another sample, so eight ranks need to be traversed up and then down the taxonomic tree. We use the EMDUnifrac implementation of the UniFrac distance^75^. An average weighted UniFrac value of 0.22 (standard deviation 0.16, minimum 0.01, maximum 0.43 and median 0.14) can be found between pairs of biological replicate samples stored under varying conditions, from the data used in ref. ^76^ and available in Qiita^77^ with study ID 10394 (35 samples matching regular expression 10394\.H1\..*(1week|fresh)). These values serve as a baseline for good (0.22) to excellent (0.01) profiling predictions with regard to this metric.

The Shannon equitability index is defined for each rank as:

$${\mathrm{Shannon}}\,{\mathrm{equitability}}\,{\mathrm{index}} = \frac{{\mathop {\sum}\nolimits_t {x_t^ \ast \times {\mathrm{ln}}\left( {x_t^ \ast } \right)} }}{{{\mathrm{ln}}\left( m \right)}},$$

where *m* is the total number of taxa *t*. The index ranges from 0 to 1, with 1 indicating complete evenness. As the diversity estimate is computed from a predicted profile alone, we assess its absolute difference to the index of the gold standard for comparison.

#### Summary statistics (all software categories)

For calculation of the summary statistics, we first scored all software result submissions in each category, that is, assembly, genome binning, taxonomic binning and taxonomic profiling, by their performance per metric on each dataset. Each result was assigned a score for its ranking (0 for first place among all methods, 1 for second place and so on). Metric results of a software submission for multiple samples of a dataset were averaged for the ranking. Taxonomic binners and profilers were ranked per taxonomic level, from domain to species, and scores computed as the sum of rankings over taxonomic levels. Over all metrics, the sum of these scores was taken as the overall summary statistic for a software result submission on a dataset (Supplementary Figs. 1, 8, 10 and 12). For exploring further, problem-specific weighted metric combinations, an interactive HTML page (Supplementary Results) allows the user to select custom weights to individual metrics and visualize the results.

### Reporting Summary

Further information on research design is available in the Nature Research Reporting Summary linked to this article.

## Online content

Any methods, additional references, Nature Research reporting summaries, source data, extended data, supplementary information, acknowledgements, peer review information; details of author contributions and competing interests; and statements of data and code availability are available at 10.1038/s41592-022-01431-4.

## Supplementary information

Supplementary Information Supplementary Text and Figs. 1–16.

Reporting Summary

Supplementary Tables Supplementary Tables 1–40.

Supplementary Data Supplementary data.

## Data Availability

The benchmarking challenge and exemplary datasets (for developers to familiarize upfront with data types and formats) are available in PUBLISSO with DOIs 10.4126/FRL01-006425521 (marine, strain-madness, plant-associated), 10.4126/FRL01-006421672 (mouse gut) and 10.4126/FRL01-006425518 (human), and on the CAMI data portal (https://data.cami-challenge.org/participate). Datasets include gold standards, assembled genomes underlying benchmark data creation, NCBI taxonomy versions and reference sequence collections for NCBI RefSeq, nt and nr (status 019/01/08). Benchmarked software outputs are available on Zenodo (https://zenodo.org/communities/cami/). Raw sequencing data for the newly sequenced and previously unpublished genomes are available with BioProject numbers PRJEB50270, PRJEB50297, PRJEB50298, PRJEB50299, PRJEB43117 and PRJEB37696. Source data are provided with this paper.

## Source data

Source Data Fig. 1 Assembler performances
Source Data Fig. 2 Genome binning average completeness, average purity, ARI and percentage of binned bp.
Source Data Fig. 3 Taxonomic binning metrics, 1% filtered and unfiltered.
Source Data Fig. 4 Completeness, purity, 2–L1 norm and 16-weighted UniFrac of taxonomic profilers at genus rank.
Source Data Fig. 5 Runtime (hours) and maximum memory usage (GB) of methods.

## Code availability

Software and scripts used for data analyses and Figs. 1–5, and summary results are available at https://github.com/CAMI-challenge/second_challenge_evaluation. Supplementary Table 2 specifies the evaluated programs, parameters used and installations options, including software repositories, Bioconda package recipes, Docker images, Bioboxes and BioContainers.
