# Supplementary material for: Critical Assessment of Metagenome Interpretation: the second round of challenges
Source: Nat Methods. 2022 Apr 8;19(4):429–40. doi: 10.1038/s41592-022-01431-4 (PMC9007738; doi:10.1038/s41592-022-01431-4)
Supplement: Supplementary file 2 — Reporting Summary [file 41592_2022_1431_MOESM2_ESM.pdf]

nature research

Corresponding author(s): Alice C. McHardy

Last updated by author(s): Jan 22, 2022

Reporting Summary

Nature Research wishes to improve the reproducibility of the work that we publish. This form provides structure for consistency and transparency in reporting. For further information on Nature Research policies, see our Editorial Policies and the Editorial Policy Checklist.

Statistics

For all statistical analyses, confirm that the following items are present in the figure legend, table legend, main text, or Methods section.

n/a	Confirmed	
☐	☒	The exact sample size (*n*) for each experimental group/condition, given as a discrete number and unit of measurement
☒	☐	A statement on whether measurements were taken from distinct samples or whether the same sample was measured repeatedly
☒	☐	The statistical test(s) used AND whether they are one- or two-sided<br>*Only common tests should be described solely by name; describe more complex techniques in the Methods section.*
☒	☐	A description of all covariates tested
☒	☐	A description of any assumptions or corrections, such as tests of normality and adjustment for multiple comparisons
☐	☒	A full description of the statistical parameters including central tendency (e.g. means) or other basic estimates (e.g. regression coefficient) AND variation (e.g. standard deviation) or associated estimates of uncertainty (e.g. confidence intervals)
☒	☐	For null hypothesis testing, the test statistic (e.g. F, t, r) with confidence intervals, effect sizes, degrees of freedom and P value noted<br>*Give P values as exact values whenever suitable.*
☒	☐	For Bayesian analysis, information on the choice of priors and Markov chain Monte Carlo settings
☒	☐	For hierarchical and complex designs, identification of the appropriate level for tests and full reporting of outcomes
☒	☐	Estimates of effect sizes (e.g. Cohen's *d*, Pearson's *r*), indicating how they were calculated

Our web collection on statistics for biologists contains articles on many of the points above.

Software and code

Policy information about availability of computer code

Data collection	Metagenomes and microbial communities simulation: CAMISIM 1.2-beta<br>Metagenome assemblers: A-STAR version CAMI2019, ABySS 2.1.5, (meta)Flye 2.4.1, 2.8, 2.8.1, GATB 1.0, (Meta)HipMer 1.0, 1.2.2, 2.0, MEGAHIT 1.1.2, 1.1.4, 1.2.7, Metahit_LINKS, MG-Atlas 2.1.0, (meta)SPAdes 3.13, 3.13.1, 3.14, OPERA-MS 0.83, 0.9, RayMeta 2.3.1<br>Sequence read quality trimming or error correction: Trimmomatic, DUK (versions unknown)<br>Genome binners: Autometa cami2, CONCOCT 0.4.1, 1.1.0, LSHVec cami2, MaxBin 2.0.2, 2.2.7, MetaBAT 0.25, 2.13-33, 2.15-5, MetaBinner 1.0, 1.1, 1.2, 1.3, MetaWRAP 1.2.3, SolidBin, UltraBinner 1.0, VAMB fa045c0, 3.0.1<br>Taxonomic binners: DIAMOND 0.9.28, Ganon 0.1.4, 0.3.1, Kraken 0.10.5 beta, 2.0.8 beta, LSHVec cami2, MEGAN 6.15.2, NBC++ b31015, PhyloPythiaS+ 1.4<br>Taxonomic profilers: Bracken 2.2, 2.6, CCMetagen 1.1.3, Centrifuge 1.0.4 beta, DUDes cami1, 0.08, FOCUS cami1, 1.5, LSHVec cami2, Metalign 0.6.2, MetaPhlAn cami1, 2.9.22, 3.0.7, MetaPhyler 1.25, mOTUs cami1, 2.0.1, 2.5.1, NBC++ b31015, sourmash gather 3.3.2, TIPP cami1, 4.3.10<br>Further software and scripts used for data analyses are available at https://github.com/CAMI-challenge/second_challenge_evaluation.<br>Supplementary Table 2 specifies the evaluated programs, parameters used, and installations options, including software repositories, Bioconda package recipes, Docker images, Bioboxes, and Biocontainers. Source data and scripts for Figures 1-5 are available online (https://github.com/CAMI-challenge/second_challenge_evaluation/).
Data analysis	MetaQUAST 5.1.0rc and 5.0.2 (metagenome assembly analysis), AMBER 2.0.3 (genome and taxonomic binning analysis), OPAL 1.0.10 (taxonomic profiling analysis)

For manuscripts utilizing custom algorithms or software that are central to the research but not yet described in published literature, software must be made available to editors and reviewers. We strongly encourage code deposition in a community repository (e.g. GitHub). See the Nature Research guidelines for submitting code & software for further information.

Data

Policy information about availability of data

All manuscripts must include a data availability statement. This statement should provide the following information, where applicable:

- Accession codes, unique identifiers, or web links for publicly available datasets
- A list of figures that have associated raw data
- A description of any restrictions on data availability

The benchmarking challenge and exemplary datasets (for developers to familiarize upfront with data types and formats) will be available in PUBLISSO with the DOIs 10.4126/FRL01-006425521 (marine, strain madness, plant-associated), 10.4126/FRL01-006421672 (mouse gut), and 10.4126/FRL01-006425518 (human) and on the CAMI data portal for download (https://data.cami-challenge.org/participate). Datasets include gold standards, assembled genomes underlying benchmark data creation, NCBI taxonomy versions, and reference sequence collections for NCBI RefSeq, nt and nr (status 019/01/08). Raw sequencing data for the newly sequenced and previously unpublished genomes are available with the BioProject numbers PRJEB50270, PRJEB50297, PRJEB50298, PRJEB50299, PRJEB43117, and PRJEB37696. Benchmarked software outputs are available on Zenodo (https://zenodo.org/communities/cami/).

Field-specific reporting

Please select the one below that is the best fit for your research. If you are not sure, read the appropriate sections before making your selection.

☒ Life sciences ☐ Behavioural & social sciences ☐ Ecological, evolutionary & environmental sciences

For a reference copy of the document with all sections, see nature.com/documents/nr-reporting-summary-flat.pdf

Life sciences study design

All studies must disclose on these points even when the disclosure is negative.

Sample size	Marine dataset: 10 samples (100 Gb); strain madness dataset: 100 samples (400 Gb); plant-associated dataset: 21 samples (315 Gb). These samples were simulated from genomes and profiles reflecting community genome abundance distributions, number of replicates, and sequencing technologies. In addition, a 688 Mb clinical pathogen dataset was used. Dataset sizes reflect common experimental designs in shotgun metagenomics studies, based on metagenome shotgun sequencing projects available in the NCBI short-read archive since 2010.
Data exclusions	No data were excluded.
Replication	Software versions and parameters were documented in Supplementary Table 2, Supplementary Text, and on Github (as described in code availability). All attempts of replication were successful.
Randomization	Not relevant to our study, as metagenomic samples of different datasets were simulated based on experimentally obtained taxonomic profiles for the specific groups (datasets representing different environments); see sections Genome sequencing and assembly and Challenge datasets in Methods.
Blinding	All genome data used for generation of the benchmark datasets and their metadata were kept confidential during the challenge and released afterwards (10.4126/FRL01-006421672). To support an unbiased assessment, program submissions were represented with anonymous names in the CAMI portal (known only to submitters), and a second set of anonymous names for evaluation and discussion in the evaluation workshop, such that identities were unknown to all except for data analysis team (F.M., Z-L.D., A.F., A.S.), and program identities revealed only after a first consensus was reached.

Reporting for specific materials, systems and methods

We require information from authors about some types of materials, experimental systems and methods used in many studies. Here, indicate whether each material, system or method listed is relevant to your study. If you are not sure if a list item applies to your research, read the appropriate section before selecting a response.

Materials & experimental systems

n/a	Involved in the study
☒	☐ Antibodies
☒	☐ Eukaryotic cell lines
☒	☐ Palaeontology and archaeology
☒	☐ Animals and other organisms
☒	☐ Human research participants
☒	☐ Clinical data
☒	☐ Dual use research of concern

Methods

n/a	Involved in the study
☒	☐ ChIP-seq
☒	☐ Flow cytometry
☒	☐ MRI-based neuroimaging
