# Supplementary material for: Critical Assessment of Metagenome Interpretation: the second round of challenges
Source: Nat Methods. 2022 Apr 8;19(4):429–40. doi: 10.1038/s41592-022-01431-4 (PMC9007738; doi:10.1038/s41592-022-01431-4)
Supplement: Supplementary file 4 — Supplementary data. [file 41592_2022_1431_MOESM4_ESM.zip › Supplementary_results.html]

CAMI 2 Challenge software rankings
